# A Novel Persistence Associated EBV miRNA Expression Profile Is Disrupted in Neoplasia

**DOI:** 10.1371/journal.ppat.1002193

**Published:** 2011-08-25

**Authors:** Jin Qiu, Katherine Cosmopoulos, Michiel Pegtel, Erik Hopmans, Paul Murray, Jaap Middeldorp, Michael Shapiro, David A. Thorley-Lawson

**Affiliations:** 1 Dept of Pathology, Tufts University School of Medicine, Boston, Massachusetts, United States of America; 2 Department of Pathology, VU University Medical Center, Amsterdam, The Netherlands; 3 Cancer Research UK Institute for Cancer Studies, University of Birmingham, Birmingham, United Kingdom; Emory University, United States of America

## Abstract

We have performed the first extensive profiling of Epstein-Barr virus (EBV) miRNAs on in vivo derived normal and neoplastic infected tissues. We describe a unique pattern of viral miRNA expression by normal infected cells in vivo expressing restricted viral latency programs (germinal center: Latency II and memory B: Latency I/0). This includes the complete absence of 15 of the 34 miRNAs profiled. These consist of 12 BART miRNAs (including approximately half of Cluster 2) and 3 of the 4 BHRF1 miRNAs. All but 2 of these absent miRNAs become expressed during EBV driven growth (Latency III). Furthermore, EBV driven growth is accompanied by a 5–10 fold down regulation in the level of the BART miRNAs expressed in germinal center and memory B cells. Therefore, Latency III also expresses a unique pattern of viral miRNAs. We refer to the miRNAs that are specifically expressed in EBV driven growth as the Latency III associated miRNAs. In EBV associated tumors that employ Latency I or II (Burkitt's lymphoma, Hodgkin's disease, nasopharyngeal carcinoma and gastric carcinoma), the Latency III associated BART but not BHRF1 miRNAs are up regulated. Thus BART miRNA expression is deregulated in the EBV associated tumors. This is the first demonstration that Latency III specific genes (the Latency III associated BARTs) can be expressed in these tumors. The EBV associated tumors demonstrate very similar patterns of miRNA expression yet were readily distinguished when the expression data were analyzed either by heat-map/clustering or principal component analysis. Systematic analysis revealed that the information distinguishing the tumor types was redundant and distributed across all the miRNAs. This resembles “secret sharing” algorithms where information can be distributed among a large number of recipients in such a way that any combination of a small number of recipients is able to understand the message. Biologically, this may be a consequence of functional redundancy between the miRNAs.

## Author Summary

miRNAs are small (∼22 bp) RNAs. They play central roles in many cellular processes. Epstein-Barr virus (EBV) is an important human pathogen that establishes persistent infection in nearly all humans and is associated with several common forms of cancer. To achieve persistent infection, the virus infects B cells and uses a series of discrete transcription programs to drive these B cells to become memory B cells – the site of long term persistent infection. It was the first human virus found to express miRNAs of which there are at least 40. The functions of a few of these miRNAs are known but their expression in latently infected normal and neoplastic tissues in vivo have not been described. Here we have profiled EBV miRNAs in a wide range of infected normal and neoplastic tissue. We demonstrate that there are indeed latency program specific patterns of viral miRNA expression and that these patterns are disrupted in EBV associated tumors implicating EBV miRNAs both in long term persistence and in oncogenesis.

## Introduction

Epstein-Barr virus (EBV), a member of the gamma-herpesvirus family, is the most common virus in the human population[1]. It infects nearly 95% of adults and persists latently for the lifetime of healthy hosts. In the generally accepted model of EBV persistence, the virus initiates infection by crossing the epithelium of the oro-pharynx and infecting resting naïve B cells in Waldeyer's ring [2], [3]. The establishment of persistent infection is characterized by the sequential employment of a series of latency transcription programs that allow the virus to drive the newly infected naïve B cell into the memory B cell compartment. Initially newly infected cells express all of the nine known latent proteins (Latency III) whose function is to cause the resting B cell to become an activated lymphoblast. This program may be important for cancer development, because it is capable of initiating the activation of B cells *in vitro* into continuously proliferating lymphoblastoid cell lines (LCL). Furthermore, some of the latent proteins have been shown to possess oncogenic, pro-proliferation and/or pro-survival functions that could contribute to the development of malignancy [4]. The activated naïve B lymphoblasts in vivo rapidly migrate to the follicle to participate in a germinal center reaction [5], [6]. Here they continue to proliferate but, unlike in vitro, they switch to a more restricted latency program (Latency II) where only three of the latent proteins are expressed. Ultimately the cells leave the germinal center as resting memory B cells (MemB) – the site of long term latent persistent infection. In MemB cells, all viral protein expression is extinguished (Latency 0) except when the cells divide and express EBNA1 (Latency 1), the protein required for replication of the viral genome. This mechanism is thought to allow EBV infected cells to escape immune surveillance, enabling lifelong persistence.

EBV was discovered in Burkitt's lymphoma (BL) and was the first human tumor virus identified. Subsequent studies revealed that the virus is associated with several other lymphoid and epithelial malignancies including Hodgkin's disease (HD), nasopharyngeal (NPC) and gastric carcinomas (GaCa) (reviewed in [1], [7]). Surprisingly, none of the tumors that arise in immunocompetent individuals uses the viral growth promoting program Latency III. Instead they use Latency I (BL) and II (HD, NPC and GaCa), Latency III specific transcription is not detected. Several lines of evidence have led to the suggestion that certain stages of the EBV life cycle are linked to the pathogenesis of distinct cancer types expressing the equivalent latency program. Thus EBV-positive BL cell has been linked with MemB cells and HD with germinal center B cells (GCB) [7].

**Figure 1:**
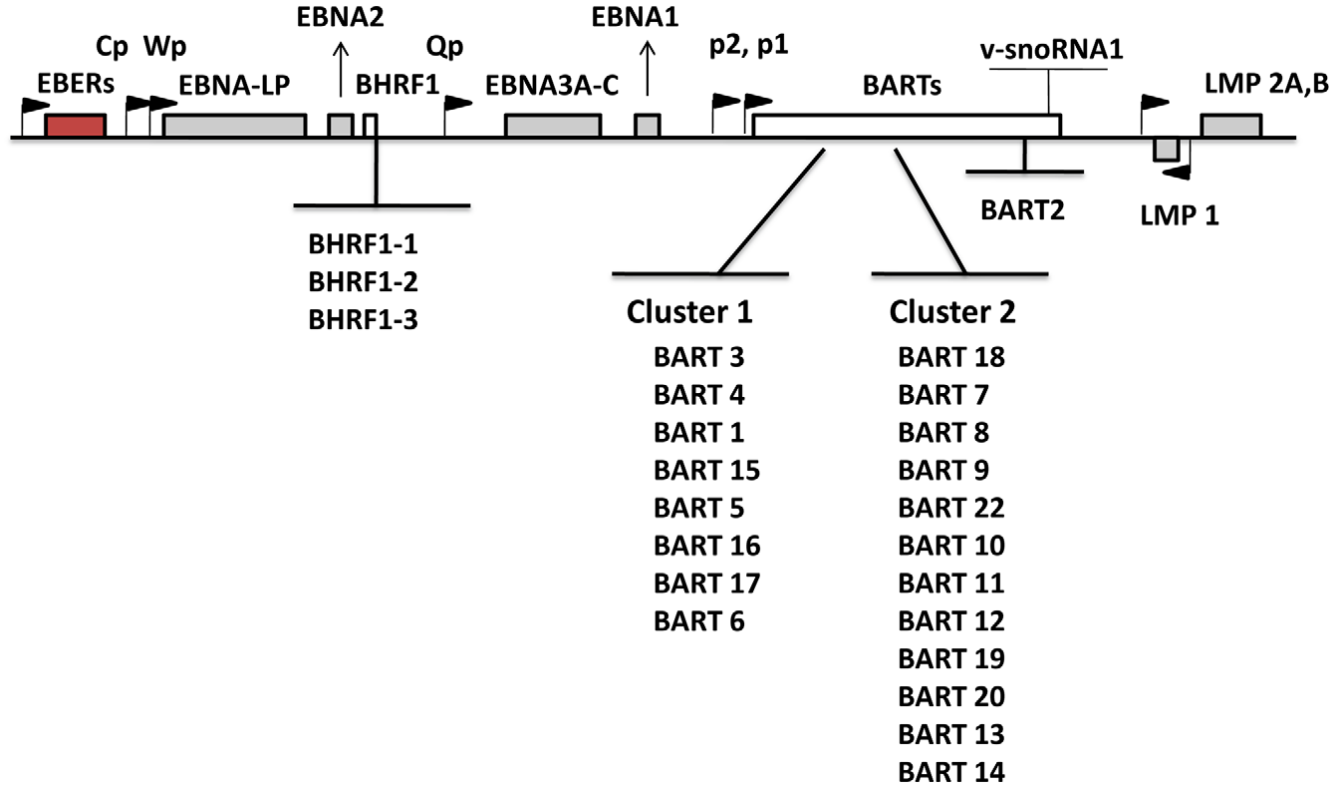
Schematic representation of the location and ordering of EBV miRNAs within the EBV genome. EBV miRNAs are derived from two transcripts: BHRF1 and BART (white bars). Precursor miRNAs are listed in the order of genomic location. The location of latent proteins (EBNAs and LMPs –grey boxes), other transcripts found in latency states (EBER – red box) and their promoters (black flags) are also shown. The V-snoRNA1[57] is also indicated at the end of the BART transcript.

Numerous studies have focused on analyzing and interpreting the function of the viral latent proteins in order to better understand their contribution to tumorigenesis. Recently, attention has been directed toward EBV microRNAs (miRNAs) that are expressed in latently infected cells. miRNAs are short noncoding RNAs, average length 22 nucleotides, that post transcriptionally regulate gene expression [8], [9]. They function either by repressing translation or inducing mRNA degradation. An increasing body of literature suggests that miRNAs are involved in a wide array of biological events. With studies using molecular biology, computational analysis and newly emerging deep sequencing techniques, 44 mature EBV miRNAs derived from 25 precursors have been described [10]–[13]. EBV miRNAs are mainly encoded from two regions: BHRF1 (Bam HI fragment H rightward open reading frame 1) and BART (Bam HI-A region rightward transcript) (Figure 1). miRNAs are the only known functional products of the BART transcripts [14]. BHRF1 derived miRNAs were reported to be highly expressed in LCL (Latency III) [10], [13], whereas BART miRNAs have been found in all EBV-infected cell lines tested including LCL, BL and NPC and tumor biopsies from NPC, GaCa and DLBCL [10], [11], [13], [15]–[18]. However a comprehensive comparative accounting is lacking since most studies only examined a limited repertoire of miRNAs (frequently with non- or semi quantitative techniques), a limited range of tissues was studied (frequently employing cell lines instead of fresh infected tissue) and appropriate computational methods for data mining were not employed. Using quantitative multiplex RT-PCR with specific 6′FAM-probes and primers for each miRNA, we have reported previously an EBV miRNA profile for NPC tissues [16] however a comparable profile does not currently exist for the other EBV associated tumors including BL, HD and GaCa. Therefore it is unknown if there is tumor specific variation in the patterns of EBV miRNA expression. A number of investigations have previously focused on the profiling of human cellular miRNAs in B-cell subpopulations and B cell associated lymphomas (for example, see [19]–[24]). However, nothing is known about the expression of EBV miRNAs in normal infected B cells in vivo and consequently it is unknown if there are specific changes in their expression associated with tumorigenesis.

Several groups have identified potential functions or targets for EBV miRNAs. It has been reported that the BHRF1 miRNAs are associated with Latency III and viral replication [15], [25]–[27], chemokine modulation [28] and cell cycle progression and proliferation [26]. There are conflicting reports about the possible role of the BARTs. They are suggested to regulate both viral [29]–[31] and cellular proteins associated with apoptosis, survival and immune evasion [32]–[34]. However, they are dispensable for infection and immortalization of B cells in vitro [35] and their absence had no reported effect on susceptibility to apoptosis of infected B cells [26]. This raises the possibility that the BART miRNAs may have an important role to play during normal infection of B cells in vivo that is not required in vitro. This parallels the behavior of LMP2 for example which is believed to play an important signaling and survival role in vivo as a B cell receptor surrogate but is completely dispensable for immortalization in vitro [36], [37].

Therefore there is an immediate need to characterize and understand the expression profiles of EBV miRNAs in normal infected B cell populations and tumors in vivo. In the present study, we aimed to quantitatively assess and computationally analyze the miRNA expression profiles in EBV-associated tumor biopsies (NPC, GaCa, BL, HD) and EBV-infected LCL, GCB and MemB cells from normal populations. The goal was to discover if subsets of miRNAs are associated with specific latency programs and if these expression profiles are disrupted during tumor development. We present the first demonstration of deregulation of EBV miRNA expression associated with tumorigenesis. Specifically, we identify a subset of BART miRNAs that are restricted to Latency III in normal infection but are up regulated in tumors that express Latency I and II.

## Results

### EBV miRNA profiling of normal and neoplastic tissues

**Figure 2:**
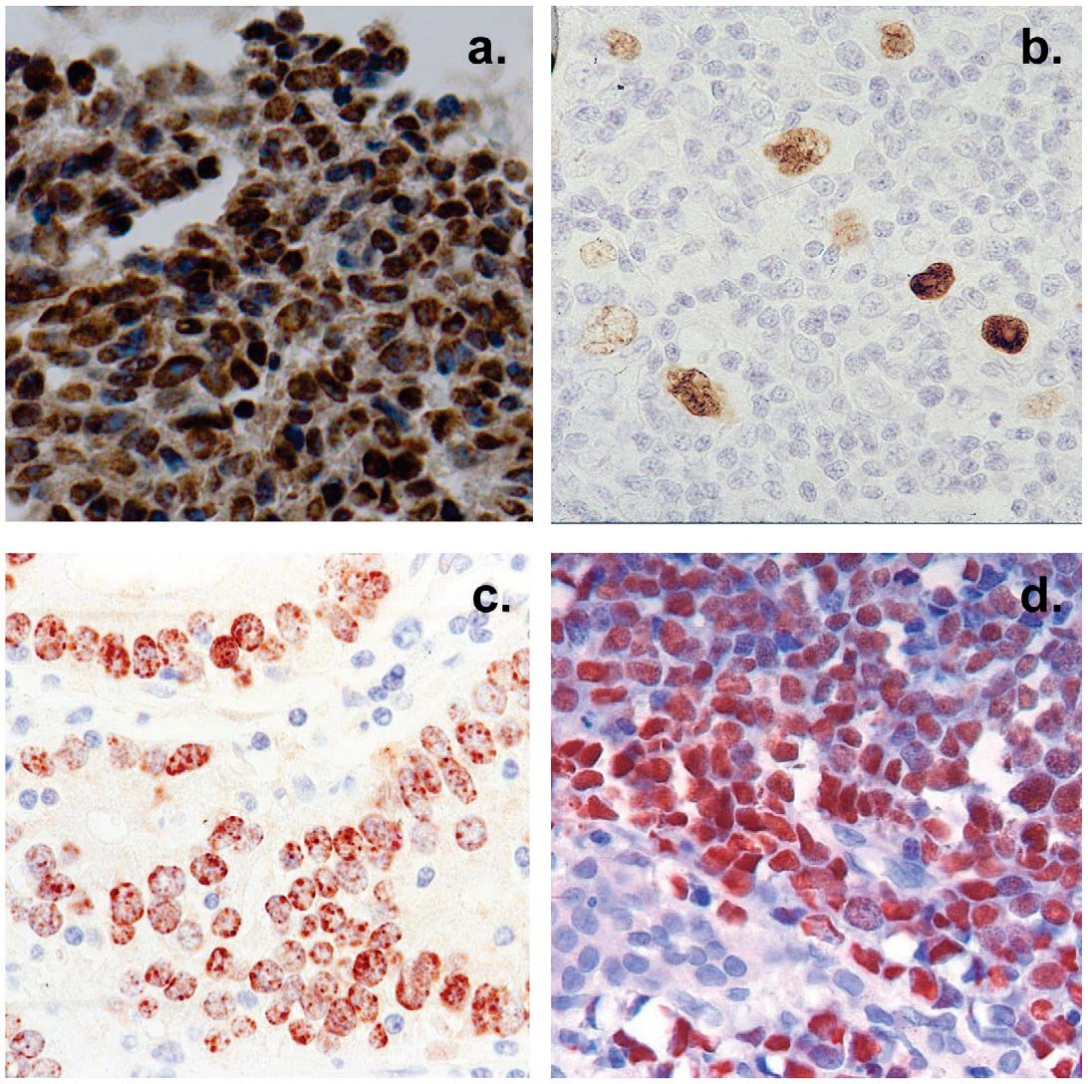
Histological cross sections of typical tumor biopsies used in this study stained for expression of EBV genes. Note that with the exception of HD less than half of the cells are non-tumor infiltrating lymphocytes. A. Nasopharyngeal carcinoma stained for the EBV nuclear antigen EBNA1. B. Hodgkin's disease stained for EBNA1 Note the sparse appearance of the tumor cells compared to the other tumor types. C. Gastric carcinoma stained for EBNA1 D. Burkitt's lymphoma stained for the EBV encoded small RNAs EBER.

**Figure 3:**
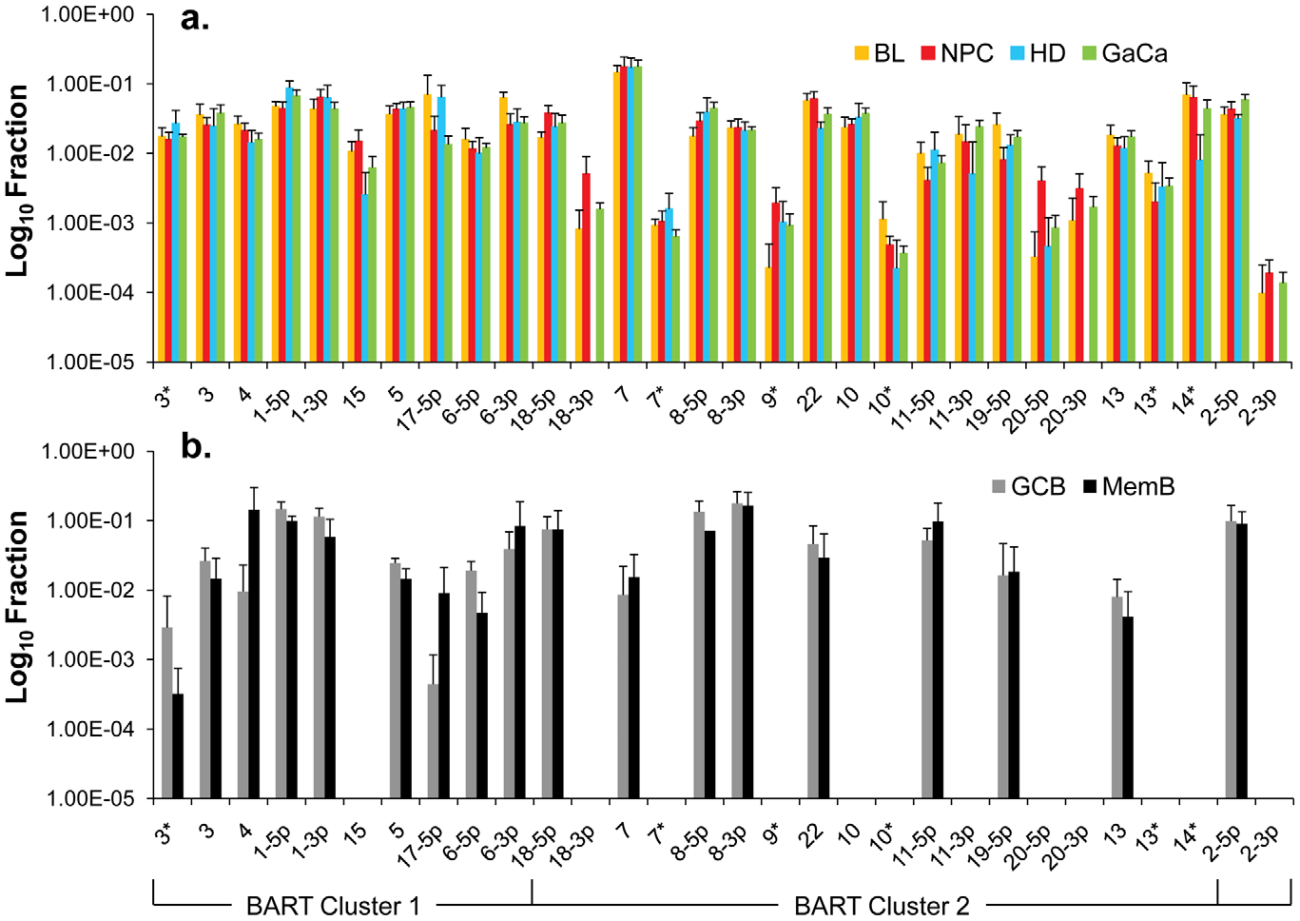
Profile of EBV BART miRNAs in normal and neoplastic tissue. The data is expressed as average and standard deviation of the fraction that each miRNA constitutes of the total of all EBV miRNAs. A. The four tumor types tested. Blue – Hodgkin's disease (n = 3): yellow – Burkitt's lymphoma (n = 6): green - gastric carcinoma (n = 6): red - nasopharyngeal carcinoma (n = 6). B. Normal infected tissue. Grey – GCB (n = 5), black – MemB (n = 4). The y intercept is a conservative approximation to the sensitivity of the assay (this will vary according to the input number of infected cells). It represents the fraction for a miRNA present at 10 copies, the lower limit of sensitivity for detection of all the miRNAs [16]. N.B. The miRNAs are presented on the x-axis from left to right in the order that they appear in the viral genome (see Figure 1).

We have previously described a technique that allows the profiling of EBV miRNAs in small amounts of EBV positive tissue [16]. We have now used this technique to profile EBV miRNA expression in primary infected normal tissues and tumor biopsies. The tissues tested are listed in Table 1. For the normal tissues, we profiled latently infected germinal center B cells (GCB, Latency II) from the tonsils (n = 5) and latently infected memory B cells (MemB, Latency I/0) from the peripheral blood (n = 4) all derived from normal, persistently infected individuals. For the tumors, we profiled four types of primary biopsies including Burkitt's lymphoma (BL: n = 6, Latency I), gastric carcinoma (GaCa: n = 6, Latency II), nasopharyngeal carcinoma (NPC: n = 5, Latency II) and Hodgkin's disease (HD: n = 3 Latency II) (Figure 2). For the tumor biopsies, we could either normalize the results to the cellular small RNA U6 or by fraction of total viral miRNAs. However, normalization to U6 was not meaningful for the GCB and MemB cells because the fraction of infected cells in these samples was both very low and variable. Therefore to compare tumor biopsies with normal infected tissue, we expressed each miRNA as a fraction of total EBV miRNAs. The results for expression of the BART miRNAs are shown in Figure 3. There were two striking findings. The first was that despite the disparate tissue origins of the biopsies and the viral latency programs they represent, the profiles for all four tumor types were remarkably similar (Figure 3A). Second, the profiles for the two normal infected tissues (GCB and MemB) were markedly different from the tumors but similar to each other (Figure 3B), despite again originating from different tissues and employing different latency program. Therefore the similarity of the profiles was determined by whether or not the tissue of origin was neoplastic not on the latency program or the tissue of origin. The most striking difference was the absence of 11 BART miRNAs from the normal tissues that are highly expressed in the tumors. These included a large subset of the Cluster 2 BART miRNAs. Of the 18 Cluster 2 miRNAs tested, all were present in the tumor biopsies but only 8/18 (44%) were found in the GCB and MemB samples. By comparison, all 10 Cluster 1 BART miRNAs were present in the tumor biopsies but only 1 was absent from the GCB and MemB samples.

**Table 1:** Tissues and cell lines used in this study.

Name	Comment
**Peripheral blood MemB cells (CD27+, CD20+)**	
MemB1	# of infected cells tested = 3.8×10^3^
MemB2	# of infected cells tested = 2.3×10^3^
MemB3	# of infected cells tested = 1.3×10^3^
MemB4	# of infected cells tested = 3.0×10^3^
**Tonsil GCB cells (CD10+, CD20+)**	
GCB1	# of infected cells tested = 6.0×10^2^
GCB2	# of infected cells tested = 1.0×10^3^
GCB3	# of infected cells tested = 6.5×10^3^
GCB4	# of infected cells tested = 4.0×10^3^
GCB5	# of infected cells tested = 3.8×10^3^
**Spontaneous LCL**	
LCL1	A.M from A. Rickinson
LCL2	Angu from A. Rickinson
LCL3	IM43 from A. Rickinson
LCL4	IM86 from A. Rickinson
LCL5	Salina from A. Rickinson
LCL6	IM82 from A. Rickinson
**Tumor Biopsies**	
HD	Hodgkin's disease
NPC	Nasopharngeal carcinoma
GaCa	Gastric Carcinoma
BL	Burkitt's lymphoma
**Tumor Cell Line**	
L591	Origin: Hodgkin's disease
C666-1	Origin: Nasopharngeal carcinoma
AGS-BX1	Origin: Gastric Carcinoma
Jijoye	Origin: Burkitt's lymphoma
Rael	“
BL36	“
Akata 2A8.1	“
Raji	“

We conclude that EBV associated tumors expressing Latency I and II up regulate a subset of BART miRNAs that are silenced in their normal infected counterparts in vivo.

### The Cluster 2 BART miRNAs absent from normal infected tissues are expressed in Latency III

**Figure 4:**
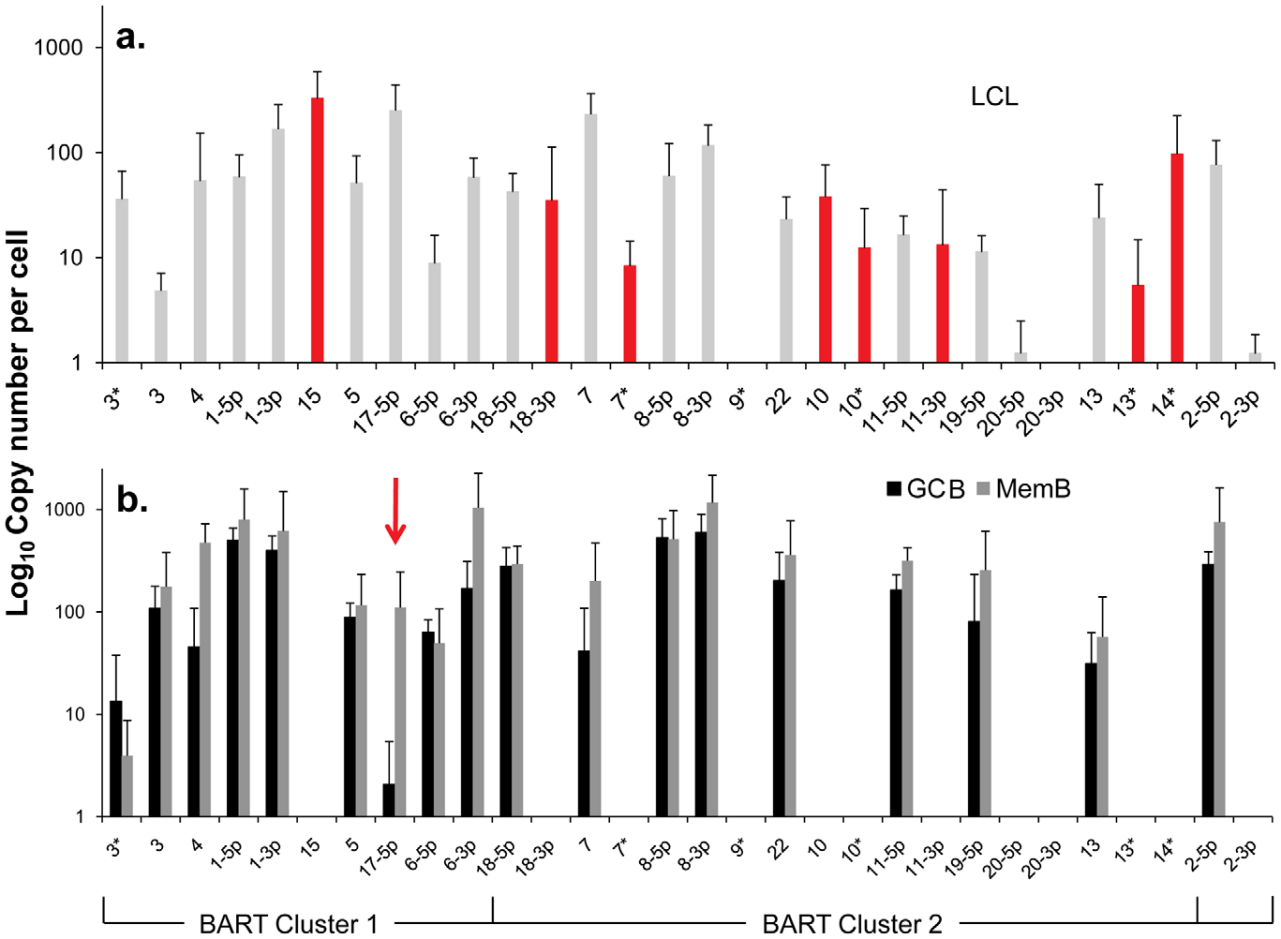
Profile of EBV BART miRNAs in vivo and in spontaneous lymphoblastoid cell lines (LCL) expressing latency III. The data is expressed as copy number of each miRNA per infected cell. A. Spontaneous lymphoblastoid cell lines LCL (n = 6). miRNAs that are significantly expressed in LCL but not GCB and MemB are highlighted in red. B. Normal infected tissue. Grey – GCB (n = 5), black – MemB (n = 4). This is the same data set as Figure 3A except now expressed as copy number /cell instead of fraction. The y intercept is an approximation to the sensitivity of detection based on the following. The maximum number of infected cells tested for GCB and MemB was 4–6×10^3^ (Table 1). Approximately 0.1% of the RNA from these samples was used for each PCR reaction i.e. equivalent to ∼5 infected cells. Since we can detect as few as 10 copies of each miRNA [16], we conclude that failure to detect a miRNA in any sample indicates it is present at a copy number ≤1/cell. The red arrow indicates miRNA 17-5p which was the only miRNA to show a striking differential expression between GCB and MemB cells. N.B. The miRNAs are presented on the x-axis from left to right in the order that they appear in the viral genome (see Figure 1).

One possible explanation for the different patterns described above is that the absent 11 BART miRNAs are associated with cellular proliferation and become down regulated when the cells enter a resting state. However, we can exclude this possibility because infected GCB are proliferating[6]. We therefore investigated an alternate hypothesis namely that these miRNAs are normally specifically expressed only in Latency III (infected lymphoblasts) i.e. with virus driven proliferation, and that their presence in tumors represents aberrant expression. To test this hypothesis we could apply a more quantitative approach since we are able to estimate absolute copy numbers per cell of the miRNAs in all three tissue types namely B cells driven to proliferate by EBV (spontaneous lymphoblastoid cell lines - LCL) derived from infected individuals (Latency III), GCB (Latency II) and MemB (Latency1/0). This was possible for the LCL because they are homogeneous cell lines. For the GCB and MemB samples we could estimate miRNA copy number per cell by first measuring the number of infected cells in the samples to be profiled and then dividing the total copy number of each miRNA by this value. The result for the BART miRNAs is shown as a bar graph in Figure 4 and the actual values are tallied in Table 2. The results for GCB and MemB were again very similar indicating that the profiles were essentially the same both in terms of relative representation (Figure 3B) and absolute copy number (Figure 4B and Table 2) for the BART miRNAs. One notable exception was 17-5p which was almost undetectable in GCB (average 2 copies/cell) but present at a copy number almost 2 logs higher in MemB (average 110 copies/cell). Of the 18 Cluster 2 miRNAs profiled 2 were undetected in all three tissues (LCL, GCB and MemB). The remaining 16 were all present in LCL. As with the tumors this included the one Cluster 1 and eight Cluster 2 BART miRNAs that were absent from GCB and MemB samples. A second feature to emerge from this comparison is that the BART miRNAs that were detected in GCB and MemB were present at copy numbers that averaged 5–10 fold higher than in LCL.

**Table 2:** Copy number per cell of EBV miRNAs in normal infected tissues and spontaneous lymphoblastoid cell lines.

miRNA§	LCL n = 6	GCB n = 5	MemB n = 4	
BHRF1-1	22	64	96	▾
BHRF1-2	34	0	0	▾
BHRF1-2*	494	0	0	▾
BHRF1-3	8	0	0	▾
2-5p	76	291	755	
2-3p	1	0	0	▾
1-5p	58	502	795	
1-3p	167	399	617	
3*	36	13	4	
3	5	108	176	
4	53	46	476	
5	51	88	116	
6-5p	9	63	49	
6-3p	58	170	1046	
15	327	0	0	▾
17-5p	250	2	110	
7	230	41	201	
7*	8	0	0	▾
8-5p	60	532	515	
8-3p	116	598	1166	
9*	0	0	0	
10	38	0	0	▾
10*	12	0	0	▾
11-5p	16	164	316	
11-3p	13	0	0	▾
13	24	31	57	
13*	5	0	0	▾
14*	96	0	0	▾
18-5p	42	279	294	
18-3p	35	0	0	▾
19-5p	11	80	255	
20-5p	1	0	0	▾
20-3p	0	0	0	
22	23	203	358	

**§:** - The miRNAs are divided into transcriptional groups. Starting from the top BHRF1, BART 2, BART Cluster 1, BART Cluster 2.

Arrows (▾) denote miRNAs detected in LCL but not (<0.1 copies/cell) the GCB or MemB cells.

We have investigated whether our failure to detect BART miRNAs from in vivo samples was due to lack of sensitivity of the PCR reaction rather than true absence. Of the 12 BART miRNAs that were not detected in GCB and MemB cells 4 were also either absent or present at marginal levels (∼1 copy /cell) in the LCL. Therefore the failure to detect these in the GCB and MemB may not be meaningful. However, the remaining 8 absent miRNAs (highlighted in red in Figure 4) were present in LCL at copy numbers ranging between 5 and 327, identical to the range in LCL for the BART miRNAs that were found in GCB and MemB (range 5–250/LCL cell). Therefore the undetected miRNAs were not consistently those present at low copy numbers in the LCL. Furthermore, when BART miRNAs were detected in GCB and MemB cells they were typically detected at higher levels/cell than in the LCL suggesting that we are not under representing or under detecting miRNAs in these samples. We also performed profiles on samples of 10^6^–10^7^ EBV negative tonsils into which had been spiked various numbers of LCL cells and on GCB and MemB samples with low numbers of infected cells. We determined that we could quantitatively profile most of the miRNAs in samples that contained ≥1000 infected cells in a population of 10^6^–10^7^ uninfected cells (data not shown). As the number of infected cells dropped from 1,000 failure of profiling tended to be associated with drop out of most or all of the miRNAs rather than selective gradual disappearance. In particular we did not see preferential drop out of the miRNAs absent from GCB and MemB. This suggests we may be approaching a general rather than miRNA specific threshold for profiling. The exceptions were mirBARTs 9, 12, 16 and 19-3p. The PCR for these miRNAs consistently gave significant signals due to cross reaction with RNA from the large number of uninfected cells and were therefore excluded from all analysis. With the exception of one GC sample (600 infected cells) all of the GCB and MemB samples assayed in our study contained >1000 infected cells (Table 1).

We conclude that there is a subset of BART miRNAs that reside predominantly in Cluster 2 and are specifically expressed only during Latency III in normal infected B cells. We refer to these as Latency III associated BARTs. Furthermore there is co-ordinate regulation of the BART miRNAs where approximately one third are extinguished and two thirds are up regulated as the cells traverse out of Latency III into Latency I and II.

### Expression level of BART miRNAs in tumors

**Figure 5:**
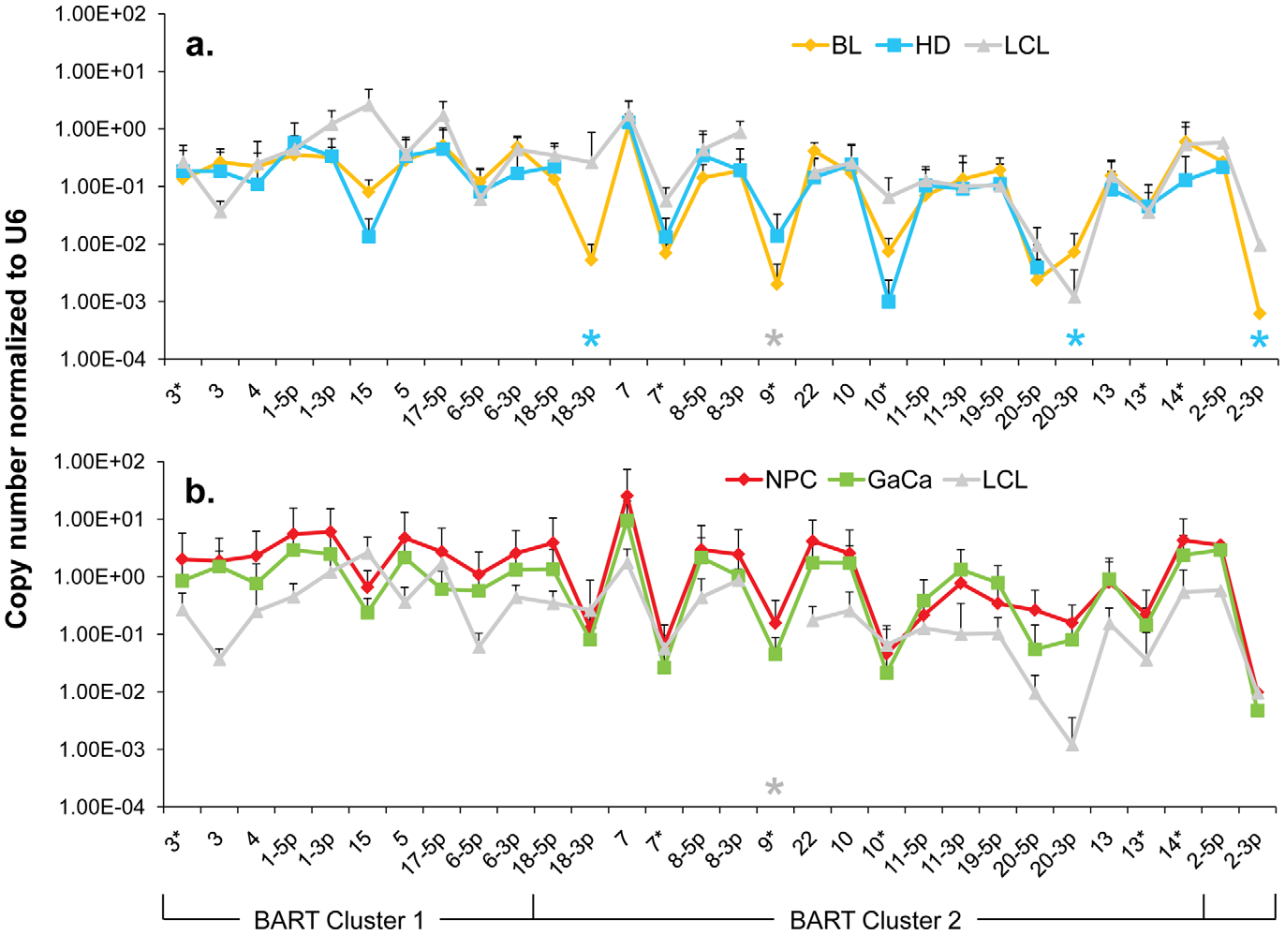
The profile of EBV BART miRNAs in LCL and neoplastic tissues. The average and standard deviation of miRNA expression after normalization to the level of expression of the ubiquitous human small RNA RNU6b is shown. The stars indicate miRNAs that were consistently absent from HD (blue) and LCL (grey) only. A. LCL versus the B cell tumors. Blue – Hodgkin's disease; yellow – Burkitt's lymphoma; grey LCL. B. LCL versus the epithelial tumors. Green - gastric carcinoma; red - nasopharyngeal carcinoma; grey - LCL. This is the same data set for the tumor biopsies as shown in Figure 3. N.B. The miRNAs are presented on the x-axis from left to right in the order that they appear in the viral genome (see Figure 1).

We have established that there is a subset of Latency III associated BART miRNAs that are also expressed in tumors, irrespective of latency type. We wished to investigate therefore whether the absolute level of expression of BART miRNAs in the tumors also matched those in the LCL i.e. lower compared to normal tissue. To gain insight into this we compared levels of BART miRNA expression in LCL and tumor biopsies after normalization to the ubiquitous small cellular RNA U6. The results are summarized in Figure 5.

Overall, the pattern of miRNA expression was similar for all of the tumors (as already shown in Figure 3A when normalized by fraction) and for the LCL. The LCL, HD and BL were closely matched (Figure 5A) both in overall pattern and copy number with the exception that the LCL lacked mirBART 9* and mirBARTs 15 and 18-3p were present at a level more than tenfold higher in the LCL. Also HD lacked mirBARTs 2-3p, 18-3p and 20-3p which were among the group of miRNAs absent from GCB and MemB. However, when LCL were compared with NPC and GaCa (Figure 5B) it was apparent that the epithelial tumors had significantly higher overall expression of the BART miRNAs. When we estimated the average fold increase of the BART miRNAs in the tumors relative to LCL, we found BL 1: NPC 13: HD 0.34: GaCa 8. Note that all of the tumors had some levels of infiltrating non-tumor cells (Figure 2) that would lower the estimates of miRNA expression. However, with the exception of HD, these constituted a small (less than half) fraction of the tumors and would not significantly affect the estimates. Therefore, the much lower levels of BART miRNAs in HD can be explained at least in part from the low abundance of tumor cells in these biopsies. Overall though it appears that the levels of BART miRNAs in the B cell tumors are comparable to those in the LCL but 5–10 fold lower than the epithelial tumors.

We conclude therefore that the BART miRNAs are expressed in all four tumors and that this represents deregulated expression of the Latency III associated BARTs. HD might represent an intermediary state where most but not all of the Latency III associated BART miRNAs are expressed.

### Expression of the BHRF1 miRNAs is not deregulated in the tumors

**Figure 6:**
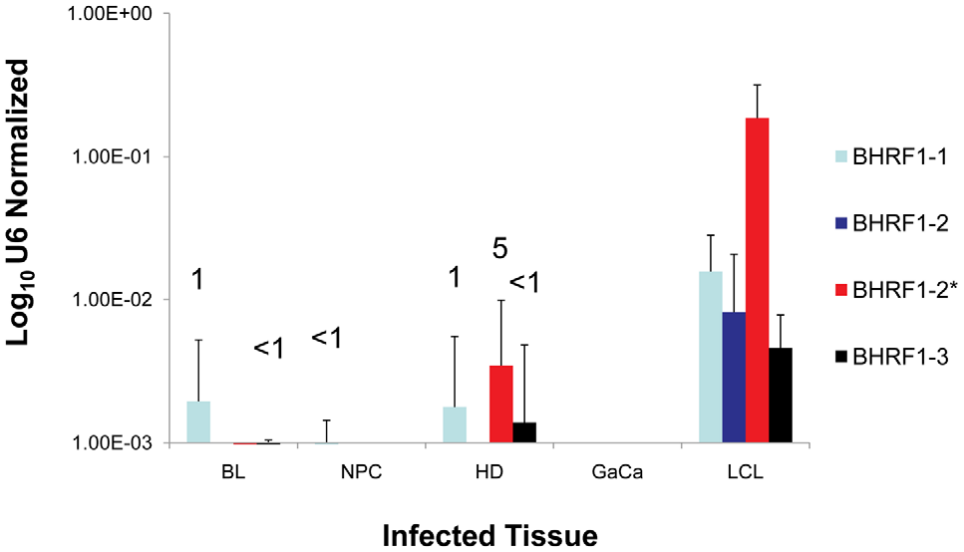
The profile of EBV BHRF1 miRNAs in LCL and neoplastic tissues. The average and standard deviation of miRNA expression after normalization to the level of expression of the ubiquitous human small RNA RNU6b is shown. The miRNAs were undetectable in GaCa. The numbers above the bars indicate estimated copy number per cell based on values from the LCL.

Previous studies have demonstrated that the BHRF1 miRNAs are expressed in Latency III where they are reported to play an anti-apoptotic role [15], [25]–[27]. This result was confirmed in our profiling (see Table 2 and Figure 6). All four BHRF1 miRNAs were readily detected in the LCL with copy numbers per cell ranging from 10–2000 depending on the miRNA and the cell line tested (Table 2 and not shown). However, they were all absent from GCB (Latency II) and only one (BHRF1-1) was found in MemB (Latency0/1).

We have demonstrated above that the Latency III associated pattern of BART miRNA expression is deregulated in all four tumor types we have studied. To test if this was also true for the BHRF1 miRNAs, we profiled their expression in all of our tumor samples and the result, compared to LCL, is shown in Figure 6. The BHRF1 miRNAs were not detected at all in GaCa and only sporadically and at low levels in the other tumor biopsies. Using the LCL values as a standard it is possible to estimate that, with the exception of BHRF1-1 in HD which was present in ∼ 5 copies, all of the rest were present at ≤1 copy per cells. This means that in the tumor samples the levels of all of the BHRF1 miRNAs were at least 10 fold lower in expression than in Latency III (LCL). Thus BHRF1 expression was not consistently deregulated in the tumors.

### EBV miRNA profiles distinguish EBV tumor types

**Figure 7:**
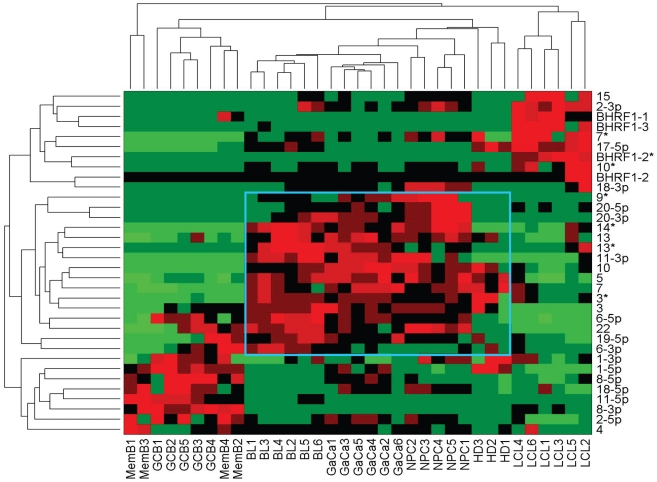
Heat map of miRNA expression in all tissues tested. The data sets used in Figure 3 and 4 were normalized based on the fraction that each miRNA comprised of the total EBV miRNA measured in each sample. A complete description is given in materials and methods. The relative up and down regulation of miRNAs is indicated by red and green respectively. Dendrograms of clustering analysis for samples and miRNAs are displayed on the top and left respectively. Tumors, forming a distinct branch on both the sample and miRNA dendrograms, are boxed in blue.

**Figure 8:**
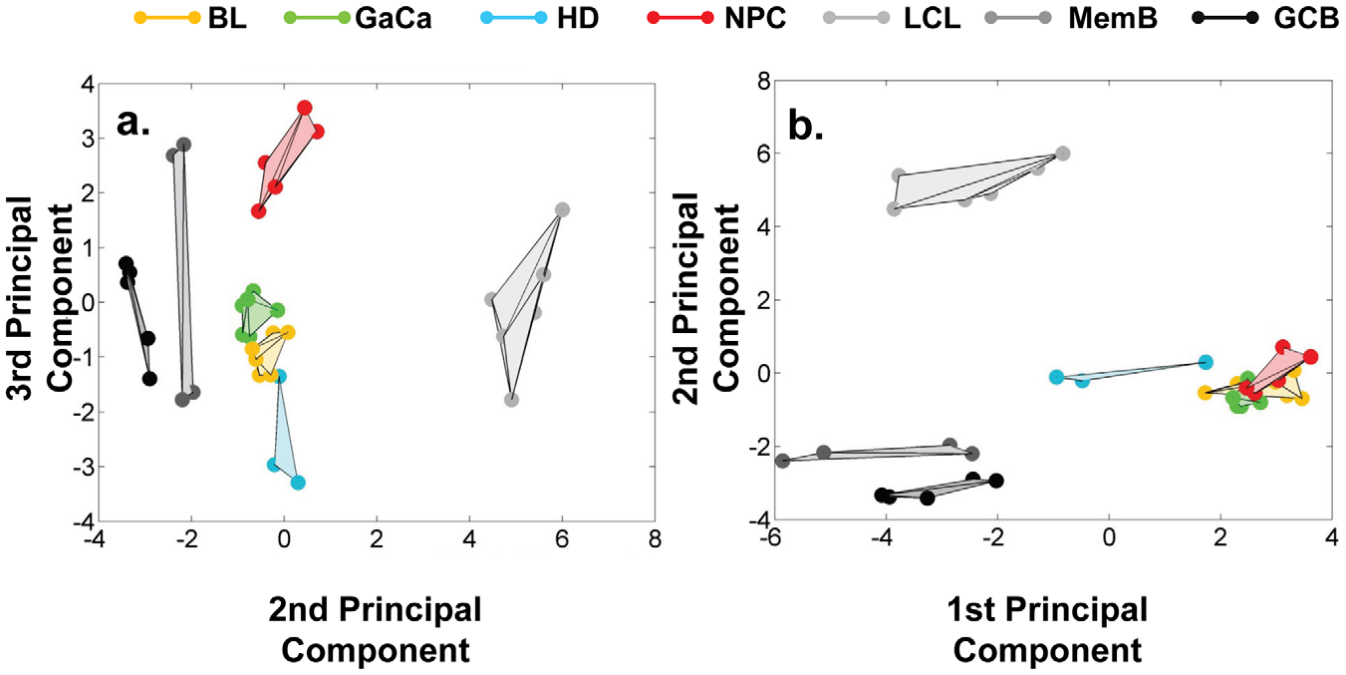
Principal component analysis (PCA) of miRNA expression in all tissues tested. PCA was performed on the same data set as shown in Figure 7. A. Second and third principal components B. First and second principal components. For a demonstration of all three components simultaneously see Video S1.

**Figure 9:**
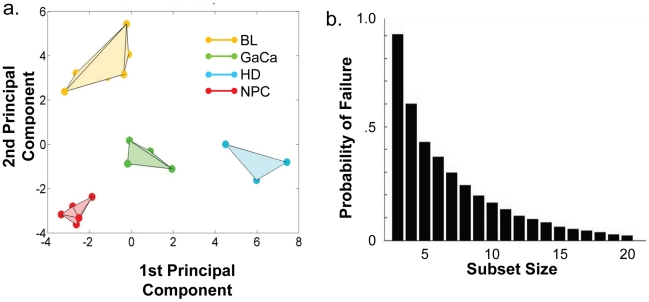
Principal component analysis of miRNA expression in all tumors tested. PCA was performed on the same data set used in Figure 3A. A. The first two principal components resolve the four tumor types. For a movie demonstrating the first three principal components see Video S2. B. Subsets containing as few as 3 miRNAs resolve the tumor types. This figure plots the failure rate of different sized sets of miRNAs. For each set size, 10,000 randomly chosen subsets were tested. N.B. this represents over sampling for the subset n = 3

We have described a pattern of deregulated BART miRNA expression in EBV associated tumors however at the crude level of our analysis we did not detect tumor type specific miRNA expression. To investigate this more rigorously we have performed clustering analysis on our data sets using heat maps and principal component analysis. Figure 7 shows a heat map with clustering dendrograms of EBV miRNA expression for all of the normal and tumor tissue samples we have tested normalized by expressing each miRNA as a fraction of the total EBV miRNAs. The ordering of the samples across the heat map coincided exactly with the three major branches of the miRNA dendrograms which in turn were associated with a functionally distinct group of samples namely normal tissue (GCB and MemB), LCL and the tumor biopsies. This analysis did not distinguish between GCB and MemB cells and the miRNAs responsible for resolving the GCB+MemB from the LCL (Table 3) coincided with those already identified above. This served as validation for the groupings indicated by the heat map. This was important because the heat map revealed new information namely that the tumors all formed a discrete branch on both the sample and miRNA dendrograms (blue box and Table 3) and that within this group the tumor samples were ordered by tumor type. This was unexpected given the similarity of the profiles as shown in Figures 3A and 5. This result was confirmed when we repeated the analysis on a second set of biopsies (not shown). This implies that there are tumor specific patterns of EBV miRNA expression. Curiously though, inspection of the heat maps, failed to identify specific subsets of miRNAs that could account for this resolution. In an attempt to understand the basis for this and possibly identify tumor specific patterns of miRNAs, we analyzed the data by principal component analysis (PCA). Figures 8 and S2 and Video S1 show the result from the same data set used for the heat map in Figure 7. Output for the first three principal components, which account for 55% of all the variation within the normalized data, is shown and confirms that the different tissue types do indeed cluster discreetly. When we examined the contributions of different miRNAs to the first 3 principal components we reproduced the findings from the heat map (Table 3, Figure S2 ). Of greatest interest was the third principal component which resolved all four tumor types. Resolution of the four tumor types could be shown even more clearly when PCA was performed on the data from the biopsies alone (Figure 9A and Video S2)and this result was confirmed when we analyzed data from a second completely independent set of biopsies (not shown). We performed two tests of the statistical significance of these results (see Materials and Methods). Both of these tests showed that successfully separating the four cancer types has a p-value of approximately 0.001.

**Table 3:** miRNAs identified by heat map as up regulated in normal infected tissue (GC and MemB) vs LCL vs tumor biopsies and their counterparts identified by PCA.

Heat map§		PCA†		
	Up regulated	PC1 + loading	PC2 + loading	PC2 - loading
LCL	BHRF1-1		BHRF1-1	
	BHRF1-2		BHRF1-2	
	BHRF1-2*		BHRF1-2*	
	BHRF1-3		BHRF1-3	
	2-3p		2-3p	
	7*		7*	
	10*		10*	
	15		15	
	17-5p		17-5p	
	18-3p		18-3p	
Normal Tissue	1-3p			
(MemB +GCB)	1-5p			1-5p
	2-5p			2-5p
	8-3p			8-3p
	8-5p			8-5p
	11-5p			11-5p
	18-5p			18-5p
				6-5p
Tumor biopsies	3	3		
	3*	3*		
	5	5		
	6-3p	6-3p		
	6-5p	6-5p		
	7	7		
	9*	9*		
	10	10		
	11-3p	11-3p		
	13	13		
	13*	13*		
	14*	14*		
	19-5p	19-5p		
	20-3p	20-3p		
	20-5p	20-5p		
	22	22		
		7*		
		18-3p		

**§:** - See Figure 6.

**†:** - See Figure 7 and Figure S2.

We developed a number of analytical tools to try and extract information about the miRNAs responsible for this effect to no avail. The reason for this became apparent when we attempted to identify which miRNAs were essential and which dispensable for resolving the four tumor types by PCA. To do this we randomly generated subsets of miRNAs and asked if they were capable of resolving all four tumor types by PCA. The surprising result, shown in Figure 9B, was that 10% of such subsets that contained just 3 miRNAs and 60% that contained 5 miRNAs could resolve the tumors. When we then looked at which miRNAs were present in these subsets we found that all of the miRNAs were represented – there was no subset of miRNAs that was uniquely responsible for distinguishing the tumors. Similarly when we asked the question: which miRNAs were dispensable in a given sub-set, i.e. could be removed without affecting resolution of the tumors, we again found that all miRNAs were essential in certain subsets (not shown). This means that the information about the EBV miRNAs which varies between the tumor types and allows their resolution is contained in part by all of the miRNAs such that when combined multiple different subsets of miRNAs contain sufficient information to distinguish the tumors. This phenomenon is reminiscent of a behavior that is well known in computer science referred to a “secret sharing” [38], [39] and represents perhaps a novel and first description of a biological system where information is so distributed across a population.

### EBV miRNA expression profiles in tumor cell lines are not representative of the tumors

**Figure 10:**
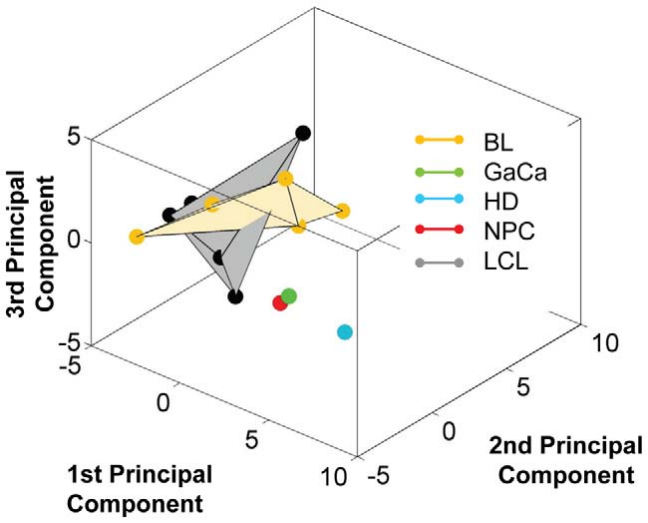
Unlike BL biopsies, the miRNA expression profile for BL derived cell lines is indistinguishable from LCL by principal component analysis. For a movie of this analysis see Video S3. Yellow – BL derived cell lines (n = 5); green; GaCa derived cell line; blue – HD derived cell line; red; NPC derived cell line; grey LCL (n = 6).

We have profiled EBV miRNAs from a number of tumor derived cells lines including 5 BL derived lines and one each of gastric, nasopharyngeal and Hodgkin's origin. When this data was analyzed in a heat map with the data from Figure 7, all of the cell lines clustered with the LCL not with the tumor biopsies they originated from (Figure S1). This was most striking for the BL lines when analyzed by PCA. As shown in Figure 10 and Video S3 the cluster of BL lines completely intersects the cluster of LCL whereas the non BL lines lie just outside. The tendency of the tumor cell lines to drift towards an LCL phenotype was further confirmed when we analyzed expression of the BHRF1 miRNAs ([16] and Table 4). As discussed above these miRNAs are associated with Latency III, the LCL phenotype, and only one is expressed at a significant level in normal infected tissue or tumor biopsies using Latency II or I. As shown in the Table they were all expressed at significant levels in the tumor derived cell lines tested with the exception of the NPC derived C666-1 cells. We conclude therefore that miRNA expression in the cell lines is not fully representative of their tumors of origin.

**Table 4:** Copy number per cell of EBV BHRF1 miRNAs in cell lines.

Cell Line	LCL	Jijoye	BL36	Rael	Raji	Akata 2A8.1	L591	AGS-BX1	C666-1
Origin		BL	BL	BL	BL	BL	HD	GaCa	NPC
BHRF1-1	22	2	126	0	227	74	114	5	0
BHRF1-2	34	88	190	154	644	210	248	27	0
BHRF1-2*	494	717	401	212	115	407	558	582	12
BHRF1-3	8	35	140	45	1	68	56	12	0

## Discussion

In this paper we have reported on the expression profiling of EBV miRNAs in a wide variety of infected normal and neoplastic tissues that express all of the known EBV associated latency transcription programs. We have shown that there are distinct patterns of miRNA expression associated with Latency III and the restricted forms of latency (Latency II/I/0) and that these patterns are deregulated in EBV associated tumors. GCB (Latency II) and MemB (Latency I/0) express the same unique, restricted pattern of miRNAs with the exception that mirBART 17-5p is preferentially expressed in MemB cells. This unique pattern includes the absence of 12 BART miRNAs that include approximately half of Cluster 2. At least 8 of these absent miRNAs are expressed in B cells expressing Latency III (LCL) and in all of the tumor biopsies we have tested although none of the tumors uses Latency III. Thus this is a Latency III restricted pattern of miRNA expression, which we refer to as Latency III associated BARTs, that is deregulated in tumors. Interestingly another group of miRNAs that are associated with Latency III, the BHRF1s [15], [25]–[27], are not deregulated in the tumors suggesting that viral miRNA deregulation in the tumors is specifically targeted at the BARTs. This is an important conclusion since it represents the first demonstration of Latency III specific gene expression in tumors that are otherwise expressing restricted (Latency I/II) forms of latency[1], [7] and raises the possibility that BART miRNAs may contribute to oncogenesis. The observation of latency program specific miRNA expression could only have been made by studying in vivo derived infected material since the Latency III associated BARTs are expressed in all tumor biopsies and cell lines we have tested.

In the generally accepted model of EBV persistence, newly infected naïve B lymphoblasts (LCL) expressing Latency III, switch to Latency II when transiting the germinal center (GCB) to become resting memory B (MemB) cells, the site of long term persistence where viral latent protein expression is extinguished [2], [3], [7]. Our results here suggest that the transit from EBV driven growth into more restricted forms of latency in GCB and MemB cells is associated with turning off expression of the Latency III associated BARTs and up regulation of the remaining BART miRNAs by 5–10 fold. This is not simply related to the cessation of proliferation because latently infected GCB are, like LCL cells, proliferating [6]. Since proliferation in the GC is not driven by Latency III, the viral growth program, we may conclude that the Latency III BART profile (presence of the Latency III associated BARTs and reduced expression of the remaining BARTs) is specifically associated with EBV driven growth. One caveat to this conclusion is that it would have been desirable to confirm the Latency III pattern of miRNA expression on in vivo infected cells rather than spontaneous LCL. Unfortunately newly infected tonsil naïve B cells in vivo are present at a level 5–10 fold lower than infected GCB [40]. This puts them below the threshold of sensitivity and reliability for our profiling and therefore was technically not feasible.

Several studies have reported on potential roles for EBV miRNAs. There is no striking correlation between these reports and the BART miRNAs we have as the Latency III associated BARTs. Marquitz et al [41] have suggested that Cluster 1 and 2 BART miRNAs interact in apoptosis resistance by targeting BIM. However, their observations are not consistent with those of Seto et al, who have reported that BART miRNAs have no impact on LCL growth or survival in vitro[26], or that the entire BART region can be deleted without impacting the transforming capacity of the virus[35] or that the prototypical laboratory strain B95-8 has most of the BART region, including most of Cluster 2, deleted yet is unimpaired in its transforming ability. The explanation for this discrepancy may lie in the fact that Marquitz et al performed their studies in an epithelial cell line not in B cells. Taken together these results suggest that the Latency III associated miRNAs we have identified, may play a crucial survival role in vivo for newly infected naïve B lymphoblasts activated by the EBV Latency III program but that this role is dispensable for in vitro growth much as has been shown for LMP2 [36], [37]. We assume that the specific up regulation of this group of miRNAs in tumors implies they could play a similar survival role in tumor development.

Our results suggest that expression of the Latency III associated BARTS is coordinately regulated. It seems unlikely that this is occurring at the level of transcription/splicing. It is known that the BART miRNAs are derived from the first four introns of the BART transcript prior to the splicing event [42] and the miRNAs absent from GCB and MemB cells are not contiguous but randomly distributed among these introns. For examples, Bart 15 is located in the region between exon 1a and 1b whereas Bart 10 and Bart 20 are in the junction of exon 2 and 3. Therefore, it is unlikely that the differential miRNA expression we have described is related to the selection of splicing patterns. Other possible mechanisms that are known to regulate miRNA expression are differential DNA methylation [43]–[45] and RNA editing both of which have been shown to function on BART miRNAs [46], [47]. However, these mechanisms defer rather than answer the question as to why or how this particular subset of miRNAs is targeted for coordinate expression. A mechanisms that we favor is based on the observation that the stability of miRNAs is dependent on the presence of their target mRNA [48]. In this case the absence of miRNAs in GCB and MemB that are present in LCL and the tumors would arise because the mRNAs targeted by those miRNAs were only present in LCL and the cancers.

We were surprised to find that the four tumor types clustered together in the heat map. This was irrespective of the tumor type, tissue of origin or the EBV transcription program that they employed. Perhaps more unexpected was our finding that despite the very similar patterns of miRNA expression the different tumor types were nevertheless clearly distinguished in two separate assays (heat map/clustering and PCA) applied to two completely separate data sets (p in both cases  = 0.001). The basis for this resolution is less clear since it is not associated with any particular subset of miRNAs. Rather we discovered that a majority of all subsets of five or more miRNAs and some subsets as small as three were capable of distinguishing all four cancer types. Even more curious was the finding that every miRNA is capable of contributing to one of these subsets. Taken together, these results mean that the signal distinguishing these cancers is highly redundantly encoded across the miRNA expression profiles. Such a distribution of information is uncommon indeed may never have been reported before for a biological system. However, this type of behavior is well known in physics and computer science where there is a close analogy to secret sharing algorithms [38], [39]. For example, it is possible to share a secret message among any number (in our case ∼40) people in such a way that if any 5 of them divulge their information to each other, the message can be read. It is interesting to speculate that the signal in our assays, i.e., the causes of or responses to each cancer type is in some similar manner parceled out among the miRNAs. How this might work at the molecular level is unclear but we assume it must reflect extensive redundancy in the miRNAs both in the number that target the same gene and in targeting genes that lie in the same or parallel signaling pathways related to tumor development. Evidence for such layers of redundancy in miRNA function is well known [49]–[52]and has recently been reported for both the Cluster 1 and 2 BART miRNAs in reducing apoptosis susceptibility in an epithelial cell line [41].

It has been suggested previously, based on cell lines, that the copy number of BART miRNAs is higher in epithelial cells than B cells. This is consistent with our estimates of the relative abundance of the miRNAs in our tumor biopsy samples versus the LCL. However, the correlation of high expression with epithelial tissue is confounded by our measurement of relative abundance in the MemB and GCB populations where the expressed BART miRNAs are present at comparable levels to the epithelial tumors. The exact meaning of these variable levels of expression is therefore now unclear.

The only EBV latency transcription program that we found to be associated with a specific pattern of miRNA expression was Latency III characterized by up regulation of the Latency III associated BART and BHRF1 miRNAs. The later serves as validation for our approach since it has been reported [27] and confirmed [15] previously. We found BHRF1 expression in some of the tumor biopsies notably BL and HD although they were present at a very low level which we estimate to be generally less than one copy per cell. The absence of BHRF1 miRNAs from EBV associated tumors is consistent with previous findings that BHRF1 miRNAs were not found in biopsies from GaCa [53] and DLBCL tissues [17]. By contrast we found abundant BHRF1 miRNAs in the tumor derived cell lines. This, together with our finding that the BART miRNA expression profile in these lines tended to resemble LCL more closely than the originating tumor biopsies, casts doubt on the value of using such lines to study EBV miRNAs.

In conclusion, we have presented the first comprehensive profiling of EBV miRNAs from in vivo derived normal and neoplastic tissue. These results demonstrate specific patterns of expression in Latency III versus more restricted forms of latency and deregulation of miRNA expression in tumors.

## Materials and Methods

### Cell lines and culture

The EBV-positive lymphoblastoid cell line (LCL) IB4 (gift of Dr. Elliot Kieff) and the murine CB59 T cell line (gift of Dr. Miguel Stadecker) were used respectively as the positive and negative control for EBV. For miRNA profiling, six spontaneously EBV-infected B LCLs (gift of Dr. Alan Rickinson) with different EBV strains (type 1 or type 2), five EBV-positive Burkitt's lymphoma (BL) cell lines Rael (gift of Dr. Sam Speck), Jijoye, BL36, Raji and Akata 2A8.1 (gift of Dr. Jeff Sample), the gastric carcinoma (GaCa) line AGS/BX1 (gift of Dr Lindsey Hutt-Fletcher), the nasopharyngeal carcinoma (NPC) line C666-1 and the Hodgkin's disease (HD)-derived cell line L591 (gift of Dr. Paul Murray) were included in this study (Table 1). The EBV-negative BL lines Akata, BJAB, DG75, BL2 and BL31, the NPC line HONE-1(gift of Dr. Ronald Glaser), and the GaCa line AGS were used as the negative controls. The GaCa cell lines were grown in Ham's F-12 medium containing 10% fetal bovine serum (FBS), 2 mM sodium pyruvate, 2 mM glutamine, and 100 IU of penicillin-streptomycin. All other cell lines were maintained in RPMI 1640 medium with the same supplements.

### Ethics statement–clinical biopsies and primary cells

The research described herein was approved by the Tufts University Institutional Review Board and our collaborating institutions. Peripheral blood mononuclear cells (PBMCs) of whole blood samples were provided by the University of Massachusetts at Amherst Student Health Service as previously described. Adolescents (ages 17 to 24 years) presenting to the clinic at the University of Massachusetts at Amherst Student Health Service (Amherst) with clinical symptoms consistent with AIM were recruited for this study. Blood was collected following the obtainment of written informed consent. These studies were approved by the Human Studies Committee at the University of Massachusetts Medical School (Worcester).

Tonsils were collected from patients 18 years of age or younger receiving routine tonsillectomies at the Tufts Medical Center at Boston, MA. Informed consent was not obtained since this was deidentified, discarded material and was deemed exempt by the Tufts University Institutional Review Board.

Tumor biopsy samples for this study were obtained from the archives of the Vrije Universiteit, Amsterdam (VU) medical center. Consent was not obtained because we used left over archival material from earlier studies (listed below). This was approved by the Medical Ethical Committee of the VU University medical center, Amsterdam, The Netherlands according to the code for proper secondary use of human tissue of the Dutch Federation of Biomedical Scientific Societies (http://www.federa.org). Burkitt's lymphoma (BL) samples were collected in Malawi from 1996–1998 under study nr. IC19-CT96-0132. Hodgkin's disease (HD) samples (all of nodular sclerosing subtype) were collected in Amsterdam from 1994–2002 under studies nr. KWF-VU1994-749 and 2001–2511 Gastric carcinoma (GaCa) samples were collected in Amsterdam from 1999–2004 under study nr. KWF1999–1990. Nasopharyngeal carcinoma (NPC) samples were collected in Indonesia during 2001–2005 in studies KWF-IN2000-02/03. A single case of HD was obtained from the Children's Hospital, Birmingham, UK, with permission from the Childhood Cancer and Leukaemia Group (CCLG) of the United Kingdom. This sample was used in accordance with Trent Research Ethics Committee REC reference number 05/MRE04/. Written consent was obtained and the sample taken under ethical approval obtained from South Birmingham Research Ethics Committee.

EBV status of tumor biopsies was assessed based on EBER1/2 in situ hybridization using commercial PNA-based hybridization probes (Dakocytomation, Glostrup, Denmark) and immunohistochemical staining for EBNA1 and LMP1 using previously described monoclonal antibodies[54], [55].

Tonsils were cut into small fragments in phosphate-buffered saline with 1% bovine serum albumin (PBSA) with razor blades. Cells were obtained by filtering the fragment suspensions through a 70 µm mesh size cell strainer. Mononuclear cells were isolated from buffy coats using the standard Ficoll-Paque Plus (Fisher Scientific) centrifugation method and saved for analysis.

### Cell staining and flow cytometry

Germinal center B (GCB) and memory B (MemB) cell populations were purified by fluorescence-activated cell sorting (FACS). Surface staining for FACS analysis was performed by standard procedures. Monoclonal antibodies against specific cell surface markers including allophycocyanin (APC)-labeled anti-CD19, phycoerythrin (PE)-anti-CD10, and fluorescein isothiocyanate (FITC)-anti-CD27 were used. GCB cells (CD19+CD10+) and MemB cells (CD19+CD27+) were sorted from tonsil and blood PBMCs, respectively as previously reported [5], [56]. For miRNA studies, 5×10^6^ to 10^7^ GCB cells were sorted for RNA isolation. Since the blood samples were small and MemB cells (CD19+CD27+) only account for 0.5-2 % of PBMCs, we sorted all the memory B cells into 10^6^ CB59 cells prior to RNA isolation. EBV miRNA profiling of CB59 cells indicated that it is appropriate to use them as filler cells since they do not generate detectable signals for any EBV miRNAs (data not shown).

### Limiting dilution and DNA real-time PCR

Determination of the EBV frequency of infected cells in purified GCB and MemB cells was done by limiting dilution and DNA real-time PCR as described previously [12]. Briefly, FACS-gated cells were sorted onto a 96-well plate with 10 replicates each of serially diluted cells. Genomic DNA was isolated by Proteinase K digestion, followed by real time Taqman DNA PCR specific for the W-repeat genome of EBV. The fraction of EBV-negative wells was calculated and the frequency of infected cells was estimated using the Poisson distribution.

### RNA extraction and multiplexed stem-loop RT-PCR

Total RNA was extracted from frozen sections or sorted cell populations with Trizol (Invitrogen). The quantity and integrity of the RNA was assessed by nanodrop and by the Agilent Bioanalyzer. EBV miRNA expression was assessed by real-time multiplex reverse transcript (RT)-PCR as previously detailed [16]. Briefly, multiple stem-loop RT primers specific for the 3′ end of each mature miRNA were mixed and applied to RT reaction, followed by Taqman PCR using primers and probes specifically assigned to each miRNA. Synthetic oligonucleotides representing all of the miRNAs were employed to generate the standard curves. The small cellular nuclear RNA U6 was used as the internal control for normalization. EBV-negative materials were used for negative controls. Profiles were performed in duplicate and repeated three times. Since, in our to-be-profiled sample pool the frequency of infected cells in 5×10^6^ to 10^7^ GCB ranged between 6–130 per 10^5^ GCB, most of the RNA was derived from cellular rather than viral origin. To test if this compromised any of the PCR assays we profiled 5×10^6^ and 10^7^ EBV negative cells. Of the 38 miRNAs tested only four (BARTs 9, 12, 16and 19-3p) gave detectable and significant signals with EBV negative cells so these were excluded from further analysis involving these cell types.

### Analytical methods

The data we have addressed here consist of copy numbers for 34 EBV miRNAs (38 for the cell lines and biopsies) from each of 43 samples. These include biopsies from four tumor types, EBV+ MemB-cells and GCB-cells from normal carriers, lymphoblastoid cell lines and tumor derived cell lines. We can consider these as a collection of 43 points in 34-dimensional space, i.e., 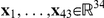. In order to perform specific comparisons, we have analyzed subsets of these points, which we will refer to generically as 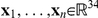. We normalized each sample to the total EBV miRNA count of that sample. That is, given a sample 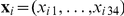


we normalized this to:
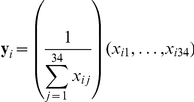



We normalized each component of the 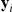 vectors by taking the Z-score for that component across all samples. That is, we took 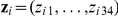 where 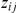 is the Z-score of 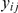 among the values 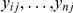.

Heat Maps We performed heat maps on 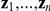 using MATLAB's clustergram function. This produces a rectangular array of squares whose rows correspond to miRNAs and whose columns correspond to samples. The color of the square denotes the relative up- or down-regulation of the miRNA in that sample. In addition, it produces dendrograms for the rows and columns which are computed using hierarchical clustering. The ordering of the rows and columns is the one most compatible with the dendrograms.

PCA. We performed PCA on 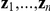 using MATLAB's pca function. We then produced 3-dimensional plots (convex hulls) by using the first 3 principal components.

Resolution and convex hulls. We are able to detect if the convex bodies are resolved or disjoint by the following computation.

Given a set of points 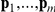, their convex hull is the set of points of the form
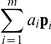



where for each *i*, *a_i_* ≥0 and
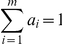



Put differently the convex hull of a set of points is the set of all possible weighted averages of these points.

Given two sets of points, 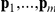 and 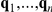, we can determine whether their convex hulls are disjoint by attempting to minimize the distance between 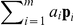 and 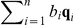 subject to the appropriate constraints. The minimum value is 0 if and only if the two convex hulls meet. We performed this constrained minimization using MATLAB's fmincon function .

The solid bodies shown in Figures 8–10 are the convex hulls of the data points grouped according to cell type. In Figure 9A they resolve the four cancer types, that is to say, these four convex hulls are disjoint. We performed two *in silico* experiments to establish a p-value for this result. Both of these involve examining large numbers of cases and determining whether the resulting convex hulls are disjoint. In the first *in silico* experiment we considered the null hypothesis that the points are randomly located in the cube. We generated sets of 20 random points in the unit 3-dimensional cube. We grouped these points into groups of 6, 6, 3 and 5 (similar to our cancer types) and determined whether the resulting convex hulls are disjoint. Note that the probability of these convex hulls being disjoint is independent of the size of the cube. Out of 10,000 trials, 12 were disjoint giving an estimated p-value of ∼0.001.

In the second *in silico* experiment we tested the null hypothesis that it is some hidden feature of the points themselves and not their grouping into the four cancer types that is responsible for the separation of the convex hulls. To test this we used MATLAB's randperm function to randomly assign the points of Figure 9A to groups of 6, 6, 3 and 5 and tested whether the resulting convex hulls were disjoint. Out of 10,000 trials, 7 were disjoint giving an estimated *p*-value of ∼0.001.

#### Digital knock-out, redundancy and the search for relevant miRNAs

To discover which miRNAs might have the most biological relevance we reasoned that these must be the ones responsible for the orderliness of Figure 9A. This led us to systematically study which subsets succeed in resolving the cancers. For a range of subset sizes *s* = 3 to *s* = 20, we generated 10,000 random subsets of size *s* drawn from the 38 EBV miRNAs appearing in our samples. (miRNA BHRF1–2* was not detected in any of the samples and was omitted from this subset analysis). Note that we have over-sampled subsets of size *s* = 3 since 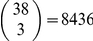. For each subset, we set to zero the data for all miRNAs not appearing in the subset and performed PCA as described above to observe if the convex hulls remained disjoint or collided. Note that occasionally MATLAB was unable to complete the PCA computation, for example, when inverting an ill-conditioned matrix leads to numerical overflow. (This occurred in less than 1% of trials, predominantly in sets of size 3). We recorded each subset and the result of the computation. We then queried this database of subsets in several ways attempting to determine which miRNAs play the largest role in resolving the cancer types.

#### Test 1: number of occurrences of each miRNA in subsets that resolve

We counted the number of times each miRNA appears across all trials in those subsets that successfully resolved the four cancer types. The result for each miRNA is derived from an arbitrary choice of 10,000 total trials of each size.

#### Test 2: number of times the addition of each miRNA converts a failing subset into a successful subset

We searched through our database of failing and succeeding subsets to discover cases in which the addition of a single miRNA converts a failing subset into a successful one.

## Supporting Information

Figure S1 Heat map of tissue samples from Figure 6 plus cell lines from all four tumor types.Cell lines are indicated by colored dots: yellow - BL lines; red - NPC line; blue – HD line; green- GaCa line.
(TIF)

Figure S2 Positive and negative loading (contributions) of the miRNAs to the 1^st^ and 2^nd^ principal components of the PCA shown in Figure 8.
(TIF)

Video S1 PCA from Figure 7 showing all three principal components.
(AVI)

Video S2 PCA from Figure 8 showing all three principal components.
(AVI)

Video S3 PCA from Figure 9 showing all three principal components.
(AVI)
