# Supplementary figures and images for: A Novel Persistence Associated EBV miRNA Expression Profile Is Disrupted in Neoplasia
Source: PLoS Pathog. 2011 Aug 25;7(8):e1002193. doi: 10.1371/journal.ppat.1002193 (PMC3161978; doi:10.1371/journal.ppat.1002193)

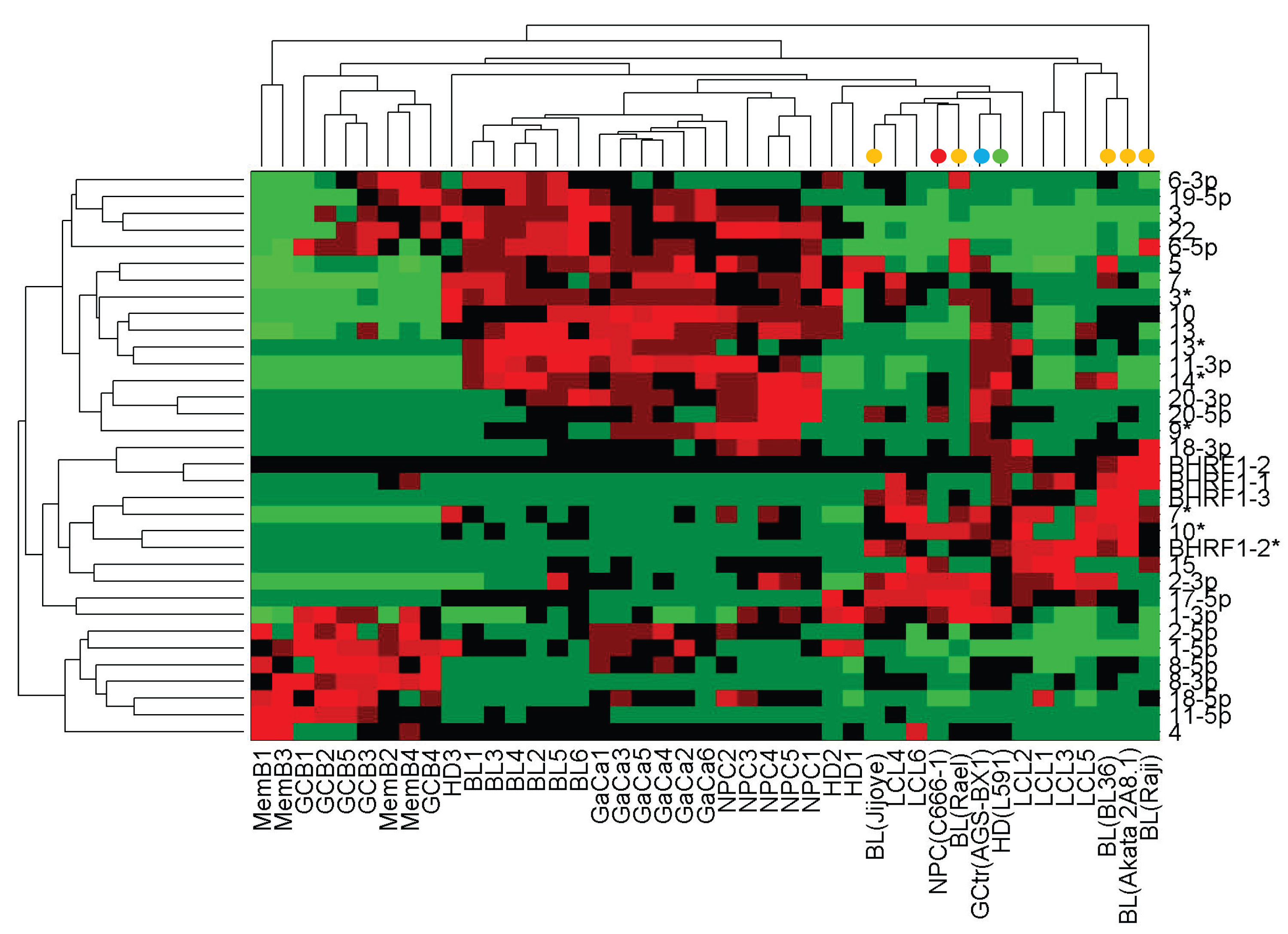

Supplement: Figure S1 — Heat map of tissue samples from Figure 6 plus cell lines from all four tumor types.Cell lines are indicated by colored dots: yellow - BL lines; red - NPC line; blue – HD line; green- GaCa line. (TIF) [file ppat.1002193.s001.tif]

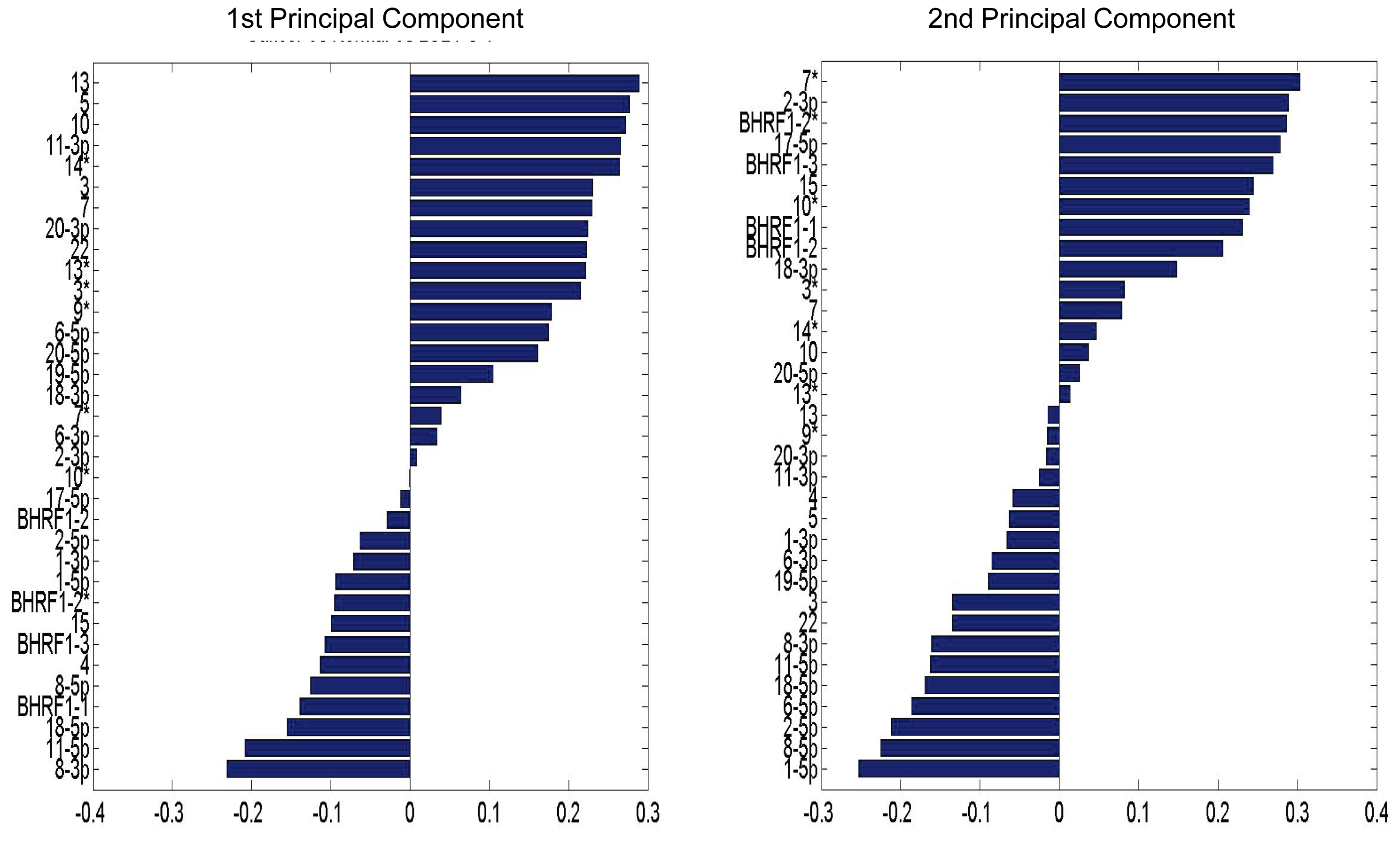

Supplement: Figure S2 — Positive and negative loading (contributions) of the miRNAs to the 1st and 2nd principal components of the PCA shown in Figure 8. (TIF) [file ppat.1002193.s002.tif]
